# Trace Amine-Associated Receptors and Monoamine-Mediated Regulation of Insulin Secretion in Pancreatic Islets

**DOI:** 10.3390/biom13111618

**Published:** 2023-11-05

**Authors:** Anastasia N. Vaganova, Taisiia S. Shemyakova, Karina V. Lenskaia, Roman N. Rodionov, Charlotte Steenblock, Raul R. Gainetdinov

**Affiliations:** 1 Institute of Translational Biomedicine, St. Petersburg State University, 199034 St. Petersburg, Russia; a.n.vaganova@spbu.ru (A.N.V.); st035112@student.spbu.ru (T.S.S.); 2 St. Petersburg State University Hospital, St. Petersburg State University, 199034 St. Petersburg, Russia; 3 Department of Medicine, St. Petersburg State University, Universitetskaya nab. 7/9, 199034 St. Petersburg, Russia; k.lenskaya@spbu.ru; 4 Department of Internal Medicine III, University Hospital Carl Gustav Carus, Technische Universität Dresden, 01307 Dresden, Germany; roman.rodionov@ukdd.de (R.N.R.); charlotte.steenblock@ukdd.de (C.S.)

**Keywords:** trace amines, trace amine-associated receptors, TAAR, dopamine, pancreatic islets, insulin, transcriptomic, GPCR

## Abstract

Currently, metabolic syndrome treatment includes predominantly pharmacological symptom relief and complex lifestyle changes. Trace amines and their receptor systems modulate signaling pathways of dopamine, norepinephrine, and serotonin, which are involved in the pathogenesis of this disorder. Trace amine-associated receptor 1 (TAAR1) is expressed in endocrine organs, and it was revealed that TAAR1 may regulate insulin secretion in pancreatic islet β-cells. For instance, accumulating data demonstrate the positive effect of TAAR1 agonists on the dynamics of metabolic syndrome progression and MetS-associated disease development. The role of other TAARs (TAAR2, TAAR5, TAAR6, TAAR8, and TAAR9) in the islet’s function is much less studied. In this review, we summarize the evidence of TAARs’ contribution to the metabolic syndrome pathogenesis and regulation of insulin secretion in pancreatic islets. Additionally, by the analysis of public transcriptomic data, we demonstrate that TAAR1 and other TAAR receptors are expressed in the pancreatic islets. We also explore associations between the expression of TAARs mRNA and other genes in studied samples and demonstrate the deregulation of TAARs’ functional associations in patients with metabolic diseases compared to healthy donors.

## 1. Introduction

Trace amines (TA) are a group of biogenic amines that were identified in mammalian brains in extremely low concentrations and were long considered neuromodulators of classical monoamine signaling [1,2]. The receptors, known as trace amine-associated receptors (TAARs), recognize both these and some other amine compounds, belong to the family of G protein-coupled receptors, and were discovered only in 2001 [3,4]. Humans possess six functional TAAR types, including TAAR1, TAAR2, TAAR5, TAAR6, TAAR8, and TAAR9 [1,2]. TAAR1 and TAAR2 sense primary amines and TAAR5–9 is tuned toward diamines, polyamines, or tertiary amines [1]. These receptors were identified at first in the olfactory epithelium, except TAAR1, which was detected primarily in the brain. Later, all TAARs were also found in the brain structures and outside the nervous system, in the immune cells, gastrointestinal tract, thyroid, and other organs and tissues [1,2,5], and their involvement in regulating immune response and metabolism is the subject of current research.

The prevalence of cardiovascular diseases (CVD), obesity, and type 2 diabetes mellitus (T2DM) is reaching epidemic proportions worldwide. One of the most significant reasons for this phenomenon is the Western diet, i.e., increased calorie intake with a predominance of saturated and trans fatty acids and insufficient physical activity. Such a kind of lifestyle leads to visceral fat accumulation and insulin resistance, which are the primary pathophysiological signs of metabolic syndrome (MetS) [6].

The term “metabolic syndrome” was introduced in 1998 by the World Health Organization to determine the condition precedent of T2DM, obesity, and related CVD. Besides excessive weight and insulin resistance, dyslipidemia, hypertension, hyperglycemia, and microalbuminuria are also associated with this state. These disorders occur together and seem to have common roots, which allows us to consider them as a syndrome [7].

MetS diagnosis is essential for public health and disease prevention. It is believed that this complex condition increases the risk of CVD, T2DM, non-alcoholic fatty liver disease, and cancer by 2–5 times compared to its individual components [8]. However, the mechanisms of MetS are not yet understood, and the search for a therapy for MetS is continuing. Currently, MetS therapy comprises lifestyle changes and pharmacological treatment of its components. Diet, physical exercise, and rejection of unhealthy habits are effective in combating MetS [9]. Metformin, statins, angiotensin-converting enzyme (ACE) inhibitors, and angiotensin receptor blockers may be prescribed to improve one or more components of MetS [10].

Dopamine (DA) is well studied as a neurotransmitter involved in movement and cognitive function regulation. It is also suggested to regulate insulin sensitivity, and lipid and carbohydrate metabolism. Recent studies have revealed the implication of DA in the pathogenesis of MetS [11,12,13]. A promising way to regulate DA signaling is its indirect modulation by TAAR1, which acts as an endogenous rheostat of DA signaling [14] and is implicated in immunity and energy homeostasis regulatory pathways [15].

TAAR1 interactions with norepinephrine (NE) [16] and serotonin (5-HT) [17] signaling were also identified. The contribution of these signaling systems in metabolic syndrome progression was demonstrated [18,19,20,21]. Pancreatic islet dysfunction is one of the metabolic syndrome development drivers [22]. Currently, the impact of DA [23,24], NE [25,26], and 5-HT [27,28] signaling on pancreatic islet function is confirmed. This review synthesizes the current evidence for the involvement of DA, NE, 5-HT, and trace amine (TA) signaling systems in regulating pancreatic islet function. Assuming that TAAR1 is the prospective therapeutic target for diseases associated with monoamine signaling disorders, we believe that TA signaling pathways should be kept in mind when developing new pharmaceutical approaches to managing metabolic syndrome.

## 2. Metabolic Syndrome and Insulin

The prevalence of MetS in the world is growing every year, varying from 9.8% to 54% depending on the criterion used [29]. However, despite the obvious social significance of MetS, it is still difficult to diagnose and treat because of the lack of a unified definition of this state. The most recognized MetS criteria are obesity, dyslipidemia, hypertension, microalbuminuria, and, especially, insulin resistance [7].

It is believed that insulin resistance is the central mechanism of the onset of MetS and related disorders, and obesity is the main manifestation of these conditions [30]. Insulin resistance is associated with a decrease in muscle and adipose cell sensitivity to insulin, while its production by the β-cells of the pancreas does not change or increase. Hyperinsulinemia is considered a compensatory attempt to overcome insulin resistance and maintain normal glucose transport into cells. However, it is contributing to metabolic, hemodynamic, and organ disorders such as T2DM and CVD, and the impairment of insulin-sensitive tissues [31].

The effects of insulin on the organs and tissues are multifaceted, which explains the heterogeneity of the MetS manifestations. The key function of insulin is the promotion of intracellular glucose accumulation by increasing cell membrane permeability, gluconeogenesis and glycogenolysis suppression, glycogenesis activation, and enhancing the synthesis of fats and proteins [32,33]. Insulin is recognized by the insulin receptor (IR), which is expressed in various organs, including the liver, heart, lung, placenta, muscle, spleen, kidney, adipose tissue, and central nervous system (CNS) [34]. IR tyrosine kinase domain autophosphorylation leads to activation of the Akt-cascade through phosphoinositol 3-kinase (PI3K) phosphorylation, which results in downstream insulin effects like decrease in gluconeogenesis and increases in glycogenesis, lipolysis suppression, and uptake of circulating glucose into cells [35]. The interaction of insulin with its receptor in the brain can activate the ERK signaling pathway [36]. Insulin together with aldosterone participates in regulating vascular stiffness, and controlling the delivery of nutrients so that insulin sensitivity failure could reduce the bioavailability of nitric oxide, and initiation of atherogenic processes like the decrease of blood flow rate and glucose removal [37].

In MetS, the transport of insulin through the blood–brain barrier is disrupted, and the level of insulin in the brain decreases at the same time as the decline of its ability to reg.ulate peripheral metabolism [38]. Because of insulin resistance, glycogen synthesis and glucose transport decrease, and lipids, free fatty acids, atherogenic triglycerides, and very low-density lipoproteins accumulate, increasing the lipolysis rate [39].

Thus, insulin resistance may be considered the most crucial prerequisite for MetS, and the prospective therapy of this condition should be targeted first at this disorder.

## 3. G Protein-Coupled Receptors and Insulin Secretion Regulation

The β-cells seem to be electrically excitable and depolarize in response to glucose and generate action potentials [40]. Commonly, β-cells uptake glucose, and when its concentration grows, the activation of glycolysis increases ATP, which mediates the inhibition of ATP-sensitive K+ channels, the opening of voltage-dependent Ca^2+^ channels, and increases cytosolic Ca^2+^ concentration with consequent insulin release [41,42]. This effect is known as glucose-stimulated insulin secretion (GSIS). Likewise, insulin secretion depends on the signals of multiple ligand–receptor systems integrated by β-cells [43]. Thus, β-cells may be classified into high or low glucose responders. The proportion of high responders depends on physiological conditions and, for example, increases during pregnancy [44].

G protein-coupled receptors (GPCRs) bind extracellular peptides or proteins, small organic molecules like free fatty acids or amino acids and their derivatives, monatomic ions, and large biological macromolecules and transmit signals to intracellular guanine nucleotide-binding proteins (G proteins). Human islets express at least 293 GPCRs, and 99 of them are the targets of either approved drugs or drugs undergoing clinical trials [41]. These receptors are critical players in GSIS paracrine regulation in β-cells by binding the glucagon, glucagon-like peptide 1, and acetylcholine derived from the α-cells which potentiate GSIS, or somatostatin (SST) originating from δ-cells and inhibiting insulin secretion [45]. Insulin, islet amyloid polypeptide (IAPP), zinc ions, and other signaling molecules are involved in GPCR-mediated autocrine regulation of GSIS in β-cells [46]. There is a plethora of other GPCR ligands that regulate islet hormone secretion like oxytocin, neurotensin, bradykinin, secretin, ATP, orexins, bile acids, fructose, fatty acids, amino acids, melanin-concentrating hormones, corticotropin-releasing hormones, and histamine [26,41,45,47,48].

The repertoire of GPCRs on pancreatic cells varies and depends on individual peculiarities and circumstances. For example, it has been demonstrated that the alpha-2C adrenergic receptor (ADRA2C) expression in mouse β-cells regularly varies depending on the circadian rhythm [49].

Pancreatic islets comprise functionally different cell types, mainly β-cells (54% of islet cells), α-cells (35%), and δ-cells (11%). The γ-cells, polypeptides (PP) cells, and ε-cells are minor cell populations in the pancreatic islets [25]. The cytoarchitecture of human islets differs somewhat from mouse islets. β-cells, α-cells, and δ-cells are scattered throughout the human islet and aligned along blood vessels. In contrast, in rodents, δ-cells are localized peripherally and β-cells are clustered together and demonstrate synchronized oscillation of membranous potential. The proportion of α-cells is significantly lower in mice compared to human islets, and their physiological impact is weaker. Together with the discrepancy in the GPCR repertoire, these differences complicate the translation of model animal studies in clinical practice [50].

Even though islets account for only 1–2% of the pancreas, they receive nearly 10% of the blood supply in this organ. The vascularization allows islets to rapidly sense the changes in nutritional or hormonal status and coordinate the insulin and glucagon secretion in the circulation. Hormone production also depends on complex autocrine and paracrine regulation, which involves not only insulin and glucagon but also δ-cell-derived SST, ε-cell-derived ghrelin, and PP synthesized and secreted in PP cells [51]. Serotonin, urocortin 3, GABA, and zinc modulate regulatory molecule secretion in α- and δ-cells [26]. All islet cell populations are thought to contribute to the function of islets through integrating systemic signals, and disturbances in these interactions could cause the development of T2DM [25].

The elucidation of monoamine-binding GPCRs’ effect on GSIS and glucose homeostasis regulation will be described below.

## 4. DA-Mediated Regulation of Insulin Secretion

### 4.1. DA Modulation of GSIS in Islets

Ingestion of a standard meal increases plasma levels of DA and its precursor L-DOPA [43]. DA infusion stimulates glucagon release and downregulates insulin secretion in healthy humans [52]. DA is found both in the endocrine and exocrine parts of the pancreas [53]. The pancreas is the main source of dopamine, which is secreted with duodenal juice and protects duodenal epithelium from damage by digestive enzymes and other harmful agents [54,55]. Recently, it was demonstrated that pancreatic acinar cells can synthesize L-DOPA, but not DA; thus, DA likely originates from nerves or endocrine cells [56].

Paracrine DA coordinates the secretory activity of the major cell groups in pancreatic islets, i.e., α-, β- and δ-cells [25]. In islets, both α- and β-cells express the catecholamine biosynthetic and signaling machinery. These cells express the components of the DA synthesis pathway, including tyrosine hydroxylase, which is the rate-limiting enzyme of this process, and aromatic amino acid decarboxylase (AADC). DA degradation enzymes monoamine oxidases MAO-A and MAO-B, catechol-O-methyltransferase (COMT), vesicular monoamine transporter, VMAT2, and the DA precursor L-DOPA were also found in islet cells [25,54,57].

**Figure 1:**
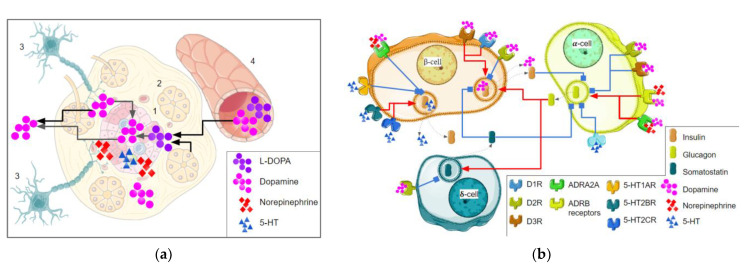
The role of monoamine neurotransmitters in the insulin secretion of β-cells. (**a**) The main sources of monoamine neurotransmitters in pancreatic islets; all monoamine neurotransmitters may be synthesized in islets (1), the dopamine synthesis in islets needs L-DOPA which is realized from acini (2) or other sources, dopamine or norepinephrine are realized from the nerve endings (3), or acquired from the circulation (4). (**b**) Monoamines and their receptors are involved in the pancreatic islet hormone production. The activation effect is marked by red arrows, inhibition is marked by blue blunt arrows, and the dotted line indicates bioactive molecules’ release. Parts of the figure were drawn by using pictures from Servier Medical Art. Servier Medical Art by Servier is licensed under a Creative Commons Attribution 3.0 Unported License.

In the β-cells, DA is packaged in the insulin granules and is released simultaneously with insulin secretion [25,42,43]. Notably, it is not only a regulatory signaling compound co-secreted with insulin. For example, insulin granules contain high concentrations of Ca^2+^, which act at neighboring β-cells to amplify insulin output via the activation of a calcium-sensing GPCR named CASR [41]. The main sources of DA in islets are summarized in Figure 1a.

The DA effect on GSIS is context dependent. DA dose-dependently inhibits insulin secretion in isolated islets in high-glucose conditions but not in a low-glucose-containing medium [58,59]. It also was demonstrated that DA regulates insulin secretion but does not modulate its production and storage in β-cells [59]. It can also suppress cell growth and initiate apoptosis in β-cells in vitro [60].

DA releases its physiological effects through interaction with two families of GPCRs, D1-like receptors (includes types 1 and 5 of DA receptors (D1R, D5R) and D2-like receptors (includes types 2, 3, and 4 of DA receptors (D2R, D3R, D4R). D1-like receptors activate G proteins of the G_α s/olf_ family, which stimulates adenylate cyclase. The D2-like receptors are coupled with G proteins of the G_α i/o_ family and inhibit adenylate cyclase [61]. In the CNS, D1-like receptors are localized mainly on the postsynaptic membranes of cells sensitive to DA, while D2-like receptors are also found on the presynaptic membranes of dopaminergic neurons, having the ability to autoregulate the release of DA [62]. DA receptors are heterogeneously expressed in different islet cell populations. In particular, D1R receptors are expressed in β -cells, D2R in α-, β-, δ-, and PP cells, D4R in β- and PP cells, and D5R in α- and δ-cells [23,24].

The D1R receptor mediates GSIS activation in β-cells, and its expression seems to prevail over the D2R expression in this cell population [52]. A D1-like receptor agonist (SKF-38393, 10 μM) stimulates GSIS, but the mechanism by which the D1 receptor regulates this process needs further investigation [23].

In contrast, D2 family activation provides an inhibitory effect on GSIS by cell depolarization [58,63]. D2R function in β-cells is not limited to participating in GSIS modulation. In vitro, the D2R antagonist domperidone protects β-cells from dedifferentiation and apoptosis and promotes the proliferation of the differentiated β-cells [64]. Recently, it was shown that D2R is involved in circadian β-cell regulation [49]. It should be noted that in the rat insulinoma cells INS-1E, D2R localization is atypical. No D2R was detected in either the cytoplasmic or the plasma membrane fractions, but it was associated with insulin granules. It remains unknown whether D2R on the granule surface is activated by extracellular dopamine only at the time of its transient contact with the cellular membrane or whether it interacts with DA in some other way [58].

Higher DA concentrations may activate ADRA2A adrenergic receptors, which also inhibit insulin secretion [25]. It is also possible that the DA effect on β-cells is indirect and mediated by its interaction with D2R on δ-cells with consequent downregulation of SST secretion. In such a way, DA activates insulin secretion [23,42]. Controversial results of some studies highlight the complexity of D2R involvement in GSIS. Knockout of D2 receptors in the insulinoma cell line INS-1 832/13 resulted in increased insulin secretion, but the global D2R knockout (KO) in mice impaired insulin secretion and caused glucose intolerance [43,65].

D3R and D4R involvement in GSIS regulation was shown in hamster β-cells. GSIS is downregulated by D3 and D4 antagonists, whereas a D3 agonist increases it [48].

Pronounced species-specific differences mark the DA-mediated regulation of α-cell glucagon secretion. DA enhances glucagon release from mouse islets, but in human islets, its effect is biphasic. DA concentrations below 1 μM reduce glucagon secretion, while higher DA concentrations enhance glucagon release. Such dualistic effects are determined by a complex repertoire of DA-binding receptors in human α-cells. D2R and D3R enable the inhibitory DA effect even in low concentrations. Higher DA concentrations lead to activation of β-adrenergic receptors, resulting in glucagon release. In mouse α-cells, β-adrenergic receptors are significantly more abundant than in humans, so the DA effect almost entirely depends on these receptors and becomes monophasic [25]. In δ-cells, D2R expression is significantly higher than in other islet cell populations and these cells seem to be the main link in dopamine-mediated GSIS regulation [23,25]. DA’s effect on pancreatic hormone secretion is briefly summarized in Figure 1b.

### 4.2. DA Signaling in CNS in the Aspect of Metabolic Syndrome

The assumption of a connection between DA homeostasis and metabolic syndrome arose when the increased risk of weight gain, hyperinsulinemia, hyperlipidemia, and diabetes mellitus, was found in patients receiving antipsychotics and other DA metabolism-targeting drugs [66]. The administration of DA receptor agonists sometimes has an ameliorative effect on the dynamics of metabolic disease recovery [67].

DA receptors are heterogeneously distributed in the mammalian CNS. D1R and D2R are commonly expressed in the striatum, nucleus accumbens, olfactory bulb, amygdala, hippocampus, substantia nigra (SN), ventral tegmental area (VTA), hypothalamus, and frontal cortex; D3R was detected in the nucleus accumbens, striatum, and cortex; D4R is expressed in the frontal cortex, amygdala, hypothalamus, and nucleus accumbens; D5R was detected in the cortex, the SN, and the hypothalamus [68,69].

D2-like receptors play the most important role in postsynaptic receptor-mediated behavioral and extrapyramidal activity [70]. Eating behavior is regulated by homeostatic signals and non-homeostatic hedonic cues both in animals and humans [71,72]. D2R contributes to the rewarding effects secondary to food intake [59], since food consumption leads to an increase in the level of DA in the brain, associated with a decrease in the expression of D2R in the nucleus accumbens in the forebrain and the ventral tegmental area in the midbrain [73]. Experimental data on mice and rats confirm the DA neurotransmission deficiency and the downregulation of D2R, DA transporter (DAT), and DA synthetic enzyme tyrosine hydroxylase (TH) under conditions of a high-calorie obesity-inducing diet [74,75]. In addition, depletion of striatal D2Rs mediates the onset of compulsive-like food seeking in rats [76,77].

The role of D2R-mediated signaling in eating behavior regulation is not restricted to its central position in the reward system. DA is released by the hypothalamic cells and tonically inhibits prolactin secretion from the pituitary through binding with D2R [78,79]. Thus, D2R antagonists may contribute to hyperinsulinemia and obesity via the control of the pituitary hormone prolactin [78].

**Figure 2:**
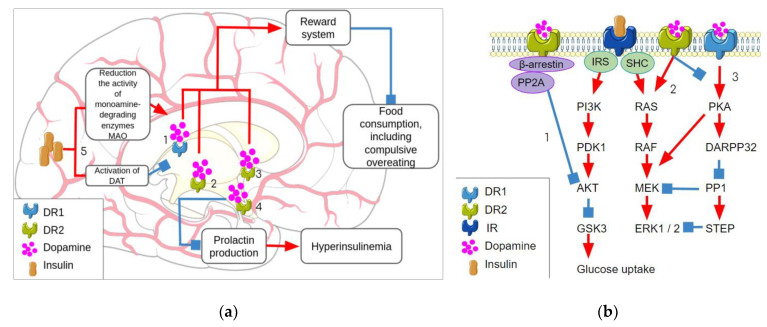
The interactions between dopamine and insulin signaling. (**a**) The crosstalk between insulin signaling and dopamine signaling in the brain where the D1R and D2R receptors in the nucleus accumbens and VTA and dopamine receptors D1R in striatonigral pathway neurons regulate the reward system, which contributes to the normalization of eating behavior (1, 2, 3). The dopamine interaction with D2R receptor in the hypothalamus prevents prolactin production and consequent hyperinsulinemia (4). At the same time, insulin dualistically affects dopamine transmission by the activation of dopamine reuptake by DAT and prevention of dopamine degradation by MAO enzymes. (**b**) Intracellular interaction of insulin signaling and dopamine signaling resulting from common downstream targets, D2R inhibits Akt signaling by the β-arrestin-mediated signaling cascade (1) and activates MEK signaling (2) while D1R stimulation activates MEK via PKA-DARPP32 pathway (3) instead. Parts of the figure were drawn by using pictures from Servier Medical Art. Servier Medical Art by Servier is licensed under a Creative Commons Attribution 3.0 Unported License. DR1—dopamine receptor 1, DR2—dopamine receptor 2, IR—insulin receptor, IRS—insulin receptor substrate, SHC—*Src* homology 2 domain containing, PP2A—protein phosphatase 2A, PI3K—phosphatidylinositol 3-kinase, PDK1—pyruvate dehydrogenase kinase 1, GSK3—glycogen synthase kinase 3, MEK—mitogen-activated protein kinase kinase, ERK 1/2—extracellular signal-regulated kinase 1/2, PKA—protein kinase A, DARPP32—dopamine- and cAMP-regulated phosphoprotein with an apparent Mr of 32,000, PP1—protein phosphatase 1, STEP—striatal-enriched protein tyrosine phosphatase.

D1R implication in the MetS phenotype is also not excluded [80]. This assumption is supported by the noticeable role of D1R in reward processing [68,69]. Furthermore, mice deficient in the SLC35D3 transporter protein exhibit MetS and obesity. SLC35D3 is expressed only in striatonigral medium spiny neurons and provides D1R transfer from the endoplasmic reticulum to the membrane [81]. The basic points of the interactions between insulin and dopamine signaling in brain structures are summarized in Figure 2a.

In humans, DA system hypofunction may be a predictor of obesity. A postmortem study of obese and non-obese patients revealed an obesity-associated decrease in DAT and TH expression without significant changes in the D2R expression level [82]. Thus, DA itself and its receptors, transporters, and synthesis enzymes are involved in eating behavior and energy balance, and their coordinated functioning breakdown may lead to metabolic disruptions.

### 4.3. Systemic Interaction of Insulin and DA Signaling Pathways 

The regulatory influence of insulin extends beyond the control of peripheral glucose levels. Insulin, along with the DA, is involved in regulating eating behavior and reward. In addition, insulin affects DA signaling, modulating the duration of DA action by regulating MAOs and DAT expression [83]. 

Insulin activates tyrosine-kinase insulin receptor IR, and its downstream signaling includes two main pathways. The insulin receptor substrate (IRS) node transduces the signal to the phosphatidylinositol 3-kinase (PI3K, a lipid kinase)/Akt (also known as PKB or protein kinase B) and the Raf/Ras/MEK/MAPK (mitogen-activated protein kinase, also known as ERK or extracellular signal-regulated kinase) pathways. Additionally, the Src homology 2 domain-containing (Shc) adapter is involved in Raf/Ras/MEK/MAPK pathway activation [84].

Overlapping insulin and DA signal transduction reflects the contribution of common downstream components in both pathways. The key point, where these pathways have crossed, is the Akt signaling. Akt is involved in a range of processes, including the control of glucose homeostasis by insulin and regulating DAT expression. Glycogen synthase kinase 3 (GSK3), which influences cell survival, circadian rhythms, mood, and cognition, is one of Akt’s downstream targets [60]. Insulin activates the Akt-signaling pathway [85]. In contrast, D2R activation blocks Akt activity through a G protein-independent beta-arrestin 2-mediated mechanism [68,86].

D2R can also trigger the extracellular signal-regulated kinase (ERK) pathway, which is involved in insulin signaling. A short-term effect of DA on D2R leads to G protein-dependent inhibition of cAMP, Ca^2+^ influx into the cell, and suppression of PKA activity. Reduction in PKA activity removes the inhibitory effect of 32 kDa DA and cAMP-regulated phosphoprotein (DARPP-32) on protein phosphatase 1 (PP1) activity, which leads to the activation of ERK. The ERK pathway mediates changes in gene expression, including DAT [68,83,87]. These interactions of two signaling pathways are represented in Figure 2b.

Metabolic diseases, in particular, T2DM, are often associated with CNS disorders. Insulin deficiency-associated disturbances in the DA system may be manifested as eating disorders, depression, and cognitive dysfunction. Insulin receptor gene KO in mice leads to the deregulation of MAO-A and MAO-B expression in the striatum and consequent increase in DA clearance time [88]. DA neuron damage in the suprachiasmatic nucleus in rats causes MetS with characteristic manifestations, including weight gain, hypertension, and insulin resistance [89].

The reduced secretory functions of pancreatic β-cells, together with complete or partial lack of insulin sensitivity, are implicated in the pathogenesis of MetS. Blockade of pancreatic D2R in mice causes glucose intolerance by decreasing insulin production and decreasing β-cells mass. The impaired β-cells replicative capacity was demonstrated in 2-month-old D2R-KO mice [65]. DA-mediated inhibition of GSIS seems to depend on the combined effect of D2 and D3 members of the D2-like receptor family and the L-DOPA transporter LAT. This transporter is expressed in islet cells as two isoforms, LAT1 and LAT2. Its concerted functioning provides effective absorption of L-DOPA in response to stimulation by the glucose supplied with food. In summary, the bioavailability of DA synthesized from dietary L-DOPA may contribute to the inhibition of GSIS [90].

Human data also support a link between DA and insulin signaling. Historically, insulin coma therapy was used in schizophrenia patients [91]. Examination of postmortem brain tissue samples from individuals with psychiatric or neurodegenerative diseases demonstrated a coordinated decrease of DA metabolism-related genes (i.e., D1R, D2R, TH, MAOB) and insulin receptor expression [92].

Considering the reciprocal interaction of the DA and insulin systems, prevention and therapy of MetS by targeting the DA metabolism may be a promising approach. Thus, D2R agonist bromocriptine has become the prospective medication to manage MetS, obesity, and T2DM. It improves glycemic parameters such as plasma cholesterol and being overweight [93]. The bromocriptine effect is mediated by both central and peripheral processes modulation. As demonstrated in animal models, it impacts the imbalance of DA signaling in the adipose tissue. Bromocriptine administration stimulates D1R and TH expression in white adipose tissue and the liver activates lipid oxidation. Additionally, bromocriptine increases insulin receptor expression levels in adipose tissue and contributes to the improvement of fasting glucose levels [94].

## 5. Norepinephrine Signaling in GSIS Regulation

Islets are innervated by adrenergic nerves projecting from the celiac ganglion or the paravertebral sympathetic ganglia. Thus, NE is released in pancreatic islets from sympathetic inputs. In addition, the murine α-cell line, alpha TC1 clone 6, may also produce NE [25]. NE reduces insulin and SST secretion and stimulates glucagon and PP secretion in islets [25,26].

Different adrenergic receptors are involved in GSIS regulation. The inhibitory effect of NE is mediated by ADRA adrenergic receptors (Figure 1a), whose blockade counteracts the inhibition of GSIS by electrical sympathetic nerve activation. GSIS inhibition by ADRA2A activation is accompanied by hyperpolarization of the β-cells and reduction in the cytoplasmic concentration of Ca^2+^ and cAMP [95]. It was found that rs553668 polymorphism in the 3′ UTR region of *ADRA2A* may be linked to impaired insulin secretion and GSIS, and an increased risk of T2DM [96].

The stimulatory action of ADRB receptors is mediated by the increased formation of cAMP in β-cells [51]. ADRB2-KO mice are glucose intolerant because of impaired GSIS; they have significantly higher blood glucose both upon fasting and under random feeding conditions and decreased fasting serum insulin levels compared to their wild-type littermates. It also was demonstrated that in 20-month-old mice, ADRB2 expression was significantly lower compared to young animals; thus, it was postulated that the reduced ADRB2 expression might contribute to the age-related decline of glucose tolerance in mice [97]. In an experimental model of T2D, the ADRB2 ligand ephedrine showed a hypoglycemic effect together with facilitating the regeneration of the pancreatic islets after their atrophy [98].

Prolonged NE exposure leads to the upregulation of insulin secretion and profound changes in the GPCR repertoire in islet cells. After chronic NE treatment of islets, the levels of G_αs_, G_αz_, G_β1_, and G_β2_ proteins were 42.8%, 19.4%, 24.8%, and 16.9% lower compared to the untreated islets, respectively. The negative regulator of insulin secretion, Ucp2, was also reduced in the NE-exposed β-cells [57].

A study of genetic factors responsible for the development of insulin resistance showed that mutations in ADRB3 may be involved in the development of tissue insulin sensitivity disorders [99]. However, this association seems to be related to NE signaling from the pancreatic islets, in particular, in visceral adipose tissue. Previously, a relationship between obesity and its associated metabolic complications, as well as increased visceral fat ADRB3 sensitivity, was proven [100].

## 6. 5-HT Participation in β-cell Modulation

5-HT regulates several functions in the CNS and outside the brain, including colon motility, immune function, and blood flow. In β-cell, 5-HT regulates GSIS (Figure 1b) and proliferation, particularly in the adaptation to hyperglycemia, a high-fat diet, or pregnancy [101]. Basal 5-HT production in β-cells is required for physiological insulin release [27]. These cells express all proteins required to synthesize and package 5-HT, which is stored in insulin granules and co-released with insulin after glucose stimulation [28,45,102,103]. Islet cells also express SERT, which allows the re-uptake of secreted 5-HT [101].

5-HT receptors are structurally different. 5-HT3Rs are ligand-gated cation channels, but all the other 5-HT receptors are GPCRs. These receptors are expressed in different populations of islet cells. β-cells express 5-HT1AR, 5-HT2CR, 5-HT1DR, 5-HT2BR, and 5-HT3Rs. 5-HT1FRs were detected in α-cells, and 5-HT5ARs are specific to δ-cells [104]. 

5-HT1AR’s agonists significantly reduced GSIS in rats [105]. In contrast, 5-HT2BR and 5-HT2CR receptors increased insulin secretion from pancreatic β-cells [43,99] through the activation of the phospholipase C pathway, with a subsequent increase in intracellular Ca^2+^ [27]. When β-cells grow in medium supplemented with 5-HT2B receptor antagonist SB204741 or high (30 mmol/l) glucose concentrations, the expression of this receptor becomes reduced [106]. It was also demonstrated that this receptor is involved in GSIS activation and β-cells proliferation in pregnancy [27,102]. The 5-HT2B receptor is a critical modulator of physiological insulin secretion and its inactivation blunts GSIS [27].

5-HT2CR expression in β-cells and its effect on GSIS activity depends on its mRNA editing. Adenosine to inosine (A-to-I) RNA editing increases the receptor 5-HT potency, agonist binding affinity, constitutive activities, and G protein coupling activity. The expression of this 5-HT2CR also depends on the feed consumption induced by an insulin resistance-initiating diet. The expression of the enzyme ADAR2 involved in the 5-HT2CR mRNA editing is also upregulated by the same diet [107].

GSIS is accompanied by 5-HT release from the β cells, and then 5-HT provides negative paracrine and autocrine feedback loops for the insulin secretion. 5-HT1ARs mediate autocrine GSIS downregulation by 5-HT on β-cells, and paracrine downregulation is implemented by the binding with 5-HT1FR on α-cells and the consequent inhibition of glucagon secretion [101,104]. In contrast, activation of 5-HT2 and 5-HT3 receptors potentiate GSIS in β-cells [101].

Downstream insulin effects may also be modulated by 5-HT. It is expected that the 5-HT2 antagonist ketanserin impairs insulin sensitivity by suppressing 5-HT2A receptor-mediated glucose uptake in skeletal muscle [108].

Several effects of 5-HT in pancreatic cells are bypassing receptors and mediated by serotonylation of proteins. In β-cells, protein serotonylation is a meaningful mechanism, thus enhancing insulin granule exocytosis by activating small GTPases [27,28].

## 7. Trace Amine Signaling and TAARs in Pancreatic Islets

### 7.1. TAARs-Dependent Regulation Mechanisms

TAs are a group of endogenous and exogenous compounds that structurally resemble biogenic monoamines. In the mammalian CNS, their concentration is extremely low and does not exceed 50 ng/g (500 nM), so the TAs were named according to their trace concentrations [1]. TAs are structurally and metabolically related to classical monoamine neurotransmitters. In low concentrations, these molecules become modulators of monoaminergic activity, especially the activities of the DA or 5-HT systems. This property determines their influence on mood and emotional background. At high concentrations, TAs demonstrate an amphetamine-like effect on the release, reuptake, and biosynthesis of catecholamines and indolamines. The ability of TA signaling to modulate monoaminergic systems attracted interest as a potential therapeutic target for the management of mental and neurodegenerative diseases [109].

The most-studied TAs are β-phenylethylamine (PEA), tyramine (TYR), tryptamine (TRP), 3-iodothyronamine (T1AM), and octopamine (OCT). These and many other less-studied mammalian TAs are TAAR agonists at their physiological concentrations. Despite 20 years of TAAR research, the progress regarding their study is relatively slow, reflecting some limitations in its exploration. First, the TAARs investigation is limited because of the low expression of these proteins. Other constraints are associated with intracellular TAAR localization and, last but not least, the wide diversity of TAAR ligands [2,110].

As is currently known, TAARs are members of the rhodopsin A-like GPCR family with seven transmembrane domains. The ligand–receptor interaction leads to cAMP accumulation, modulation of PKA and PKC signaling, and, possibly, β-arrestin 2-dependent pathway activation (at least after TAAR1 binding with its ligands). The influence of TAAR1 on the opening of internally (inwardly) rectifying K+ GirK channels was also revealed [17]. At least 26 TAAR subtypes belonging to 9 families were described in vertebrates, and their distribution in different species varies significantly. The TAAR gene family members which were identified in human and several animal models following the Ensembl database [111] are summarized in Table 1. Although TAAR expression was initially found in pancreatic β-cells, the studies of these receptors were for a long time focused specifically on their function in the CNS. This interest was attributed to the TAAR gene localization on the human chromosome 6q23.2 locus associated with susceptibility to schizophrenia [3].

The first discovered and most studied of the TAARs is TAAR1. In the brain, its expression is concentrated in the monoaminergic systems, including the VTA, the dorsal raphe, and their projections in the striatum, amygdala, hypothalamus, and frontal cortex, suggesting a TAAR1 role in modulating DA, 5-HT, and possibly NE neurotransmission [1,110,112]. 

All TAARs except TAAR1 are represented in the olfactory epithelium, where they are involved in the detection of various innate olfactory stimuli mediated by volatile amines [113,114]. However, growing evidence confirms that TAARs are also present in the CNS, particularly in limbic brain areas receiving olfactory input [5], and can be involved in adult neurogenesis, as was demonstrated in TAAR5-KO mice [115,116]. TAAR5 was found in the amygdala, arcuate nucleus, ventromedial hypothalamus, frontal cortex, hippocampus, and SN [2,117]. Systemic transcriptomic analysis demonstrated a wide representation of TAAR5 in the adult human brain, especially in the limbic brain regions associated with the processing of olfactory signals and control of emotions [117]. A TAAR6 transcript was found in the amygdala, frontal cortex, hippocampus, striatum, and SN [2,118,119].

TAAR expression has also been demonstrated outside the CNS, in the mammalian digestive, endocrine, cardiovascular, and immune systems. The expression of TAAR1, TAAR2, and TAAR6 was shown in the cells of the gastrointestinal tract. TAAR1 is predominately expressed in the mouse, rat, or human stomach and small intestine. TAAR2, TAAR6, and TAAR9 expression were detected in the mucous cells of the duodenum in mice and rats [2,120]. Human leukocytes express TAAR1, TAAR2, TAAR5, TAAR6, TAAR8, and TAAR9 [121]. Of particular interest is the TAAR expression in the islet β-cells. TAAR1 co-localization with insulin in pancreatic β-cells, as well as the modulating effects of TAAR1 on DA transmission, may underlie the relationship between TA-mediated signaling pathways and metabolic diseases, in particular, T2DM [122].

### 7.2. Relationship between the Trace Amine System and the Monoaminergic Systems

Given the involvement of the DA system in the pathogenesis of MetS, it is important to understand the possibilities of its targeting in metabolic diseases. It is known that TAAR1 modulates the DA neurotransmission regulating the rate of dopaminergic neurons’ excitation, the sensitivity of D2R to ligands, and the activity of the DAT [123]. The mechanisms underlying the interaction of the DA and TA systems will be considered. TAAR1 and D2R have a functional and structural relationship. Both receptors belong to the GPCR family and could form a heterodimer on the surface of the cell membrane [124,125]. The interaction of two receptors in the complex impacts its properties. After heterodimerization with D2R, TAAR1, which is predominantly intracellular in the monomeric form, migrates to the cell surface. In parallel, D2R signaling activity shifts from the β-arrestin 2 signaling pathway to Gi activation. The β-arrestin 2 affinities to D2-like receptors decrease, which leads to the suppression of GSK3β signaling [1,126,127]. Furthermore, after the heterodimerization, D2R and TAAR1 become co-internalized upon any of the receptors’ stimulation [124,125].

Currently, the evidence suggests an antagonistic relationship between the DA and TA systems. D2R blockade selectively enhances TAAR1 signaling [128], while TAARs’ agonists, such as PEA and TYR, inhibit the excitation of DA neurons in the VTA, SN, and nucleus accumbens. The supposed mechanism of DA neuron inhibition is related to alterations in D2R autoreceptor activity [129]. In contrast, in TAAR1-deficient mice, the DA metabolite, homovanillic acid accumulates extracellularly in the dorsal striatum and nucleus accumbens, which suggests the increased DA release in these structures. DA release is also stimulated by TAAR1 antagonists and inhibited by TAAR1 agonists in wild-type mice. However, TAAR1 insufficiency does not affect the kinetics of DA uptake and DAT function [128,130]. 

Thus, the TA system, in particular TAAR1, apparently functions as a negative control mechanism for DA release and exerts an inhibitory effect on DA neurons.

There is some evidence for the interaction of TA and 5-HT signaling. For example, TA may also indirectly cause increased susceptibility to oxidative stress in tissue through their modulation of 5-HT. The absence of TAAR1 in model animals is associated with increased DA and 5-HT signaling [131,132]. TAAR5-KO mice also showed a deregulated 5-HT and DA system [5,115]. Since it was suggested that TAAR1 could act as a rheostat of 5-HT signaling [1,131], the relation between TAARs and 5-HT signaling components is expected.

### 7.3. Background and Prospects for the Clinical Use of Trace Amines and their Receptors

The involvement of the TA system in regulating biogenic amine-mediated processes suggests its role in MetS pathogenesis. Besides the modulating effect of TAAR1 activation on DA transmission, TAARs may be implicated in the control of energy metabolism, nutrition, eating behavior, body weight, and insulin sensitivity [2]. TAAR expression outside the CNS is interesting in terms of MetS pathogenesis and therapy. TAAR1 expression was detected in the stomach and intestinal neuroendocrine cells, and in pancreatic β-cells which could impact insulin production and systemic function of the endocrine pancreas [122,133]. It was suggested that TAAR1 activation in β-cells by ractopamine can stimulate GSIS [134]. Also, it was demonstrated that the acute TAAR1 activation in vivo by the selective small molecule agonist RO5166017 stimulates insulin secretion, but in sub-chronic treatment by supplying RO5166017 with food for 7 days improves insulin sensitivity and reduces insulin plasma levels in mice [122].

The gradually accumulating evidence for TAARs’ involvement in energy metabolism makes it possible to consider these receptors as the targets in MetS therapy. In the aspect of MetS, it is important to consider the TAARs’ role in regulating eating behavior, the violation of which may underlie the etiology of obesity, which is an important factor in MetS development. A TAAR1 agonist, RO5256390, was demonstrated to prevent the compulsive eating of delicious food. The proposed background of this effect, as with drug abuse and addiction, is the modulation of DA projections in the medial prefrontal cortex. In conditions associated with disturbed eating behavior, i.e., overeating and obesity, an increased DA release during food intake occurs. Rats receiving the TAAR1 agonist RO5256390 exhibited a selective reduction in sugary food consumption without effect on either baseline intake or food restriction-induced overeating on the standard chow diet [135].

TAARs expressed outside the CNS also may be potential medication targets for MetS, as TAAR-expressing cells outside the brain also may be involved in the pathogenesis of metabolic diseases. This perspective is attractive since such TAAR-targeted compounds may release their therapeutic potential without impacting the cognitive or behavioral functions if they have a limited capacity to cross the blood–brain barrier [2]. For example, TAAR1 expression in the SST-producing D-cells in the stomach was found [136]. SST released from D-cells has a paracrine effect on the ghrelin production in the stomach and consequent downregulation of the ghrelin synthesis [137]. Ghrelin is known as the “hunger hormone” and its depletion leads to decreased food consumption. The TAAR1 agonist RO5166017 stimulates SST production in stomach D-cells and this effect seems to be TAAR1 dependent [136]. Also, it was identified that TAAR1 gene knockout in mice is associated with an increase in catabolic reaction, although the basis of this association is not described yet [138].

TAAR1, TAAR2, and TAAR9 receptors are expressed in the mucosal layer of the duodenum in mice. These receptors are suggested to be involved in hormonal secretion, or motility regulation [2,120]. In the duodenum, TAARs are co-localized with the cells producing the neuroendocrine marker chromogranin A or hormones involved in appetite regulation and glucose level control as GLP1 and PPY [122].

**Figure 3:**
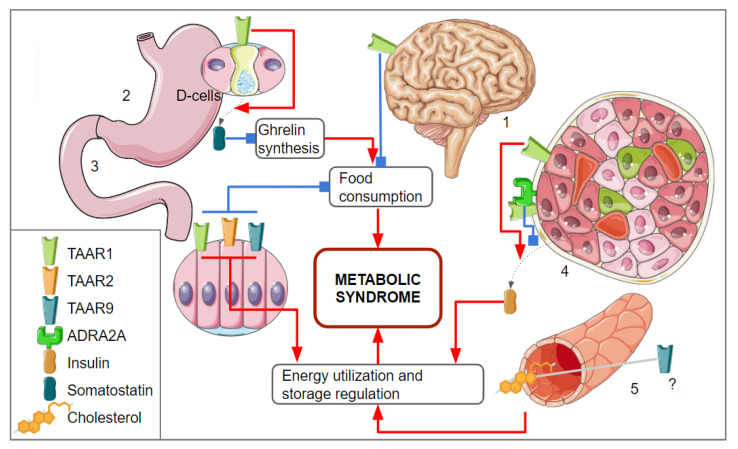
The associations between TAAR-mediated signaling and mechanisms involved in metabolic syndrome development. (1) TAAR1 expression was identified in brain structure involved in the reward system regulation which is involved in food consumption regulation; (2) TAAR1 was identified in the stomach epithelium, its activation on the D-cells leads to the somatostatin realizing, downregulation of ghrelin production, and reduces the feeling of hunger; (3) in the duodenum, TAAR1, TAAR2, and TAAR9 were identified; it is suggested that these receptors are involved in the regulation of neuroendocrine secretory cells that control appetite and glucose metabolism; (4) TAAR1 is expressed in β-cells and regulates insulin secretion, TAAR1 agonists could stimulate insulin secretion, but if TAAR1 dimerizes with ADRA2A, the effect of its activation on insulin secretion becomes inhibitory; (5) TAAR9 seems to be involved in the cholesterol metabolism regulation, but this regulatory mechanism is not clearly understood yet. Parts of the figure were drawn using pictures from Servier Medical Art. Servier Medical Art by Servier is licensed under a Creative Commons Attribution 3.0 Unported License.

The inhibition of certain TAARs may improve MetS severity. Two TAAR9-KO rat strains have lower low-density lipoprotein cholesterol levels in the plasma compared to their wild-type counterparts [139]. A high LDL-C level is a significant risk factor for CVD development. The extremely low LDL cholesterol levels may also be related to adverse effects like hemorrhagic strokes, dementia, depression, hematuria, and cancers [140]. However, TAAR9-KO rats had LDL plasma levels only twice lower than the wild-type animals, so this TAAR9-KO rats feature seems to be safe for health. No health-damaging consequences of TAAR9 deletion were found in both rat knockout lines tested [139]. The identified associations between TAARs and the regulation of mechanisms involved in metabolic syndrome development are represented in Figure 3.

Recently, TAAR1 missense mutations, S49L, and I171L were found in obese/overweight patients with slightly impaired glucose homeostasis, and R23C substitution was identified in a patient with a complete loss of insulin production. These alleles are rare, thus their impact on glucose metabolism currently has not been fully investigated. An in vitro assay revealed a partial loss of function for S49L and a complete loss of function for R23C [141].

However, it was demonstrated that the overrepresentation of tryptamine and phenethylamine producers in the enteric microbial community may play a pathogenic role in insulin resistance development. It was evidenced that the identified effect of dysbiosis is mediated by the TAAR1 activation that impairs insulin signaling in the metabolic tissues, including white adipose tissue (WAT), liver, and skeletal muscle. Thus, the TAARs, at least TAAR1, impact on the metabolic disorders seems to be dualistic [142]. Also, TAAR1 agonist N-methyltyramine provokes insulin resistance in adipocytes in vitro, despite its weak insulin-like effect in these cells in insulin-free conditions [143].

Because of the complex MetS nature, involving the disturbance of glucose and lipid metabolism accompanied by hypertension, activation inflammatory pathways, and different organ complications, multi-component treatment regimens are administered in this condition. This resulted in low patient compliance with the difficult-to-apply regimen and additional undesirable drug–drug interactions. The hopeful way to overcome these disadvantages is the development of multi-targeted therapeutic compounds that could improve different individual MetS symptoms at the same time [144,145]. The commonly known examples of such medications are PPAR agonists because PPARγ plays a significant role in the regulation of lipid metabolism, glucose homeostasis, adipogenesis, and several inflammatory processes [144].

The TAAR1 interaction with D2R with downstream behavioral effects seems to be only the “tip of the iceberg” in terms of its complex function. TAAR1 is suggested to be constitutively active or tonically activated in the central nervous system and modulate monoamine neurotransmission activity [142]. TAAR1 was found in cells involved in innate and adaptive immune response, thrombocytes, lung tissue, the esophagus and stomach, uterine cervix, prostate, kidneys, liver, and others. The function of TAARs in these cells largely remains unknown [1,15,146,147]. So, TAARs seem to be an attractive drug target as they are modulators of multiple processes in different organs and tissues.

### 7.4. TAAR Activity in the Pancreatic Islets as Part of the Systemic Metabolism Regulation by TAs

First, the idea of TA implication in metabolic processes arose when it was found that the TAAR1 agonist RO5263397 exerted potential antipsychotic activity with no metabolic side effects and minimized the weight gain in rodents undergoing D2R-blocking antipsychotic treatment [148]. Recent Phase II clinical trials with the first-in-class antipsychotic not affecting D2R, TAAR1 agonist with 5-HT1A activity SEP-363856 (Ulotaront), supported these observations [149]. In a 6-month open-label study, Ulotaront demonstrated significant antipsychotic activity without extrapyramidal-related adverse effects, a low liability for adverse weight and metabolic effects, and no effect on prolactin levels [150].

TAAR1 is expressed in the β-cells [133,151]. TAAR activation affects insulin secretion through cAMP signaling pathways. It was demonstrated that TAAR1, TAAR3, and TAAR4 ligands increase the level of cAMP and GSIS in the INS-1 mouse β-cell line [151]. Furthermore, TAAR family gene expression in islets is downregulated by glucolipotoxicity [151,152]. In β-cells, in glucolipotoxic conditions, TAAR1 expression loss is accompanied by the downregulation of stimulatory G_αs_ protein, whereas inhibitory Gαi protein becomes overexpressed. These expression changes are suggested to be associated with the suppression of GPCR-mediated GSIS upregulation [152]. 

The effect of TAAR1 activation in β-cells is the most studied TAAR-dependent process in islets. This receptor mediates GSIS potentiation by membrane depolarization, increases vesicle priming and fusion at docking sites, and stimulates phosphorylation of the key transcription factor CREB that promotes β-cell functioning. Also, TAAR1 activation could improve β-cell mass in vitro [153]. Furthermore, TAAR1 agonist RO5256390 induces potentiation of GSIS, possibly by the activation of calcium channels on the plasma membrane, which was demonstrated in the Ins-1 β-cell line in vitro [151]. 

TAAR1, TAAR2, and TAAR5 ligand T1AM and, especially, its synthetic analog SG2 with high affinity to TAAR1, affect energy metabolism, inducing ameliorative effects on insulin secretion and glucose homeostasis [154]. SG2 is also considered a promising medication in the therapy and prevention of MetS and demonstrates more suitable pharmacological properties than T1AM [150]. In contrast, GSIS stimulation by T1AM through TAAR1 binding may be demonstrated only in insulinoma cell lines [133,155]. Simultaneously, T1AM is an agonist of other receptors, including TAARs, such as TAAR5 and TAAR8, so its influence on insulin secretion may be mediated by some other targets as well [153]. On the other hand, T1AM injection stimulates hyperglycemia [156] and increases plasma glucagon levels in mice, but these effects seem to be rather dependent on the Gi/o coupled Alpha-2A adrenergic receptor (ADRA2A) than TAAR1, which is coupled with coupled Gs [157]. Another TAAR1 agonist, TYR, activates islets’ pericytes, decreases blood flow, lowers plasma insulin levels, and increases plasma glucagon levels [158]. The effect of TYR is considered to be associated with its sympathomimetic properties [154].

## 8. Evidence for TAARs Involvement in Islets Functioning from Transcriptomic Data

### 8.1. Whole Islet Transcriptomic Data

Publicly available transcriptomic data seem to be a prospective source of information about expression patterns and their systemic analysis. In this context, the Gene Expression Omnibus [159] database was searched for RNA-seq-generated transcriptomic data for human pancreatic islets. The relevant whole-tissue datasets included in the analysis are listed in Table 2. For the estimation of *TAAR* expression, all data were CPM-normalized by the edgeR R package [160] version 3.42.4. A value of 0.5 CPM was applied as the threshold for positive expression. As a result, all *TAARs*, except *TAAR2*, were detected in human islet whole-tissue samples, at least in one of the studied datasets. 

**Figure 4:**
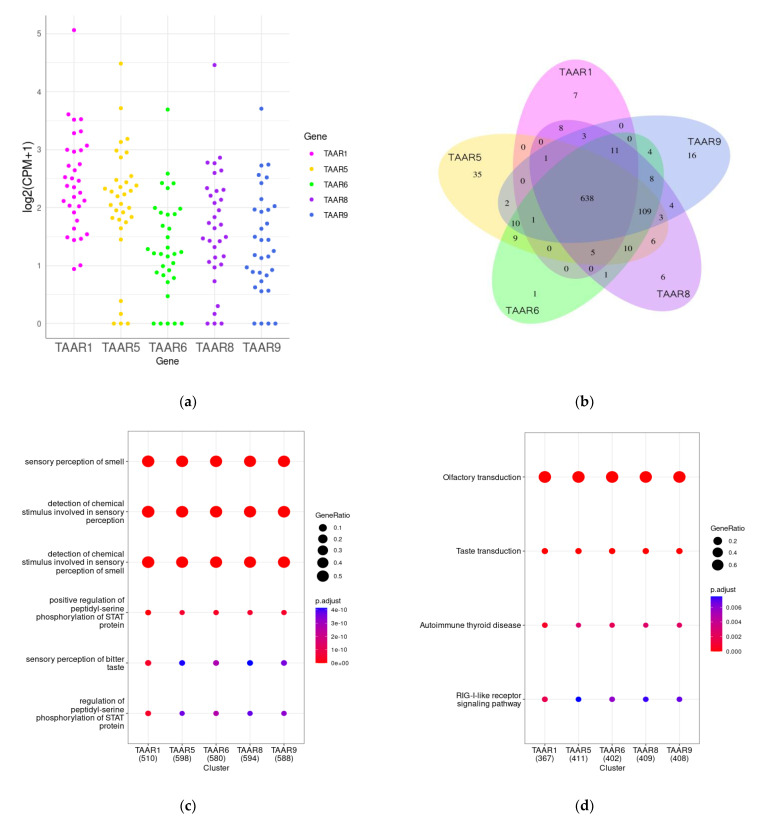
*TAARs* and *TAAR* co-expressed gene clusters in pancreatic islets. (**a**) Expression levels of *TAAR1*, *TAAR5*, *TAAR6*, *TAAR8*, and *TAAR9* in pancreatic islets isolated from healthy donors; (**b**) Venn diagram representing overlaps between *TAAR1*, *TAAR5*, *TAAR6*, *TAAR8*, and *TAAR9* co-expressed gene clusters; (**c**) analysis of Gene Ontology (GO) enrichment of *TAAR* co-expressed gene clusters; (**d**) analysis of KEGG pathway enrichment of *TAAR* co-expressed gene clusters.

Because the dataset GSE165121 was the most informative for *TAAR* expression in the pancreatic islets, we selected it for the study of the functional significance of *TAAR* expression in these structures. In this dataset, all human TAARs’ mRNA except TAAR2 were identified in most studied islet samples (Table 2, Figure 4a,b). *TAARs*’ co-expressed genes in this dataset were further analyzed to explain the significance of TAAR expression in healthy human pancreatic islets. 

*TAARs*’ co-expressed genes were selected by Pearson correlation analysis (r > 0.7, *p* < 0.05). Intriguingly, the expression levels of all detected TAARs were correlated with the expression of the same genes. *TAAR*-associated gene clusters were analyzed by the Gene Ontology (GO) terms enrichment test (i.e., identification of the gene’s functional groups that are over-represented in the gene set) and the KEGG pathway enrichment test. The analysis was performed by the clusterProfiler R package [161] version 4.8.3. Biologic process (BP) ontology was applied for the GO terms enrichment analysis.

Both applied methods reveal the co-expression of *TAARs* with genes implicated in other GPCR-mediated biological processes, particularly with olfactory and taste receptor activity, and perception of other chemical stimuli (Figure 4c,d). The co-expression of TAARs with other chemosensory receptors may mirror their implication in the islet hormone secretion regulation in the response to chemical stimuli. For example, it was demonstrated that ectopic odorant receptors (ORs) in pancreatic islets regulate insulin and glucagon secretion, fatty acid oxidation, lipogenesis, thermogenesis, and other processes. At least 47 ORs have been proven to be expressed in human pancreatic islets and the pancreatic β-cell line MIN6 [162], where these receptors regulate insulin secretion and inflammation [163,164,165,166,167]. The olfactory marker protein (OMP) is also expressed in pancreatic islets, especially in α-cells, in which it participates in glucagon secretion regulation. Taste receptors are identified in pancreatic islets [168,169]. Sweet taste receptors TAS1R1, TAS1R2, and TAS1R3 stimulate insulin secretion from β-cells [170,171,172,173].

### 8.2. TAAR1 Co-Expression Cluster Changes in Metabolic Diseases

**Figure 5:**
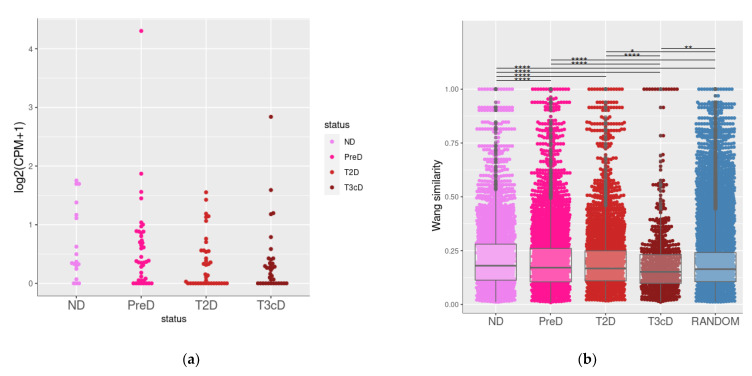
*TAAR1* and *TAAR1* co-expressed gene clusters in pancreatic islets isolated from healthy donors and patients with metabolic diseases. (**a**) Expression levels of *TAAR1* healthy donors and patients with metabolic diseases; (**b**) functional similarity of *TRAA1* co-expressed genes in islets isolated from healthy donors and patients with metabolic diseases. ND—non-diabetic (healthy donors), PreD—pre-diabetic, T2D—type 2 diabetes, T3cD—type 3c patients. *—*p* < 0.05, **—*p* < 0.01, ****—*p* < 0.001

The GSE164416 dataset includes gene expression data for islets of healthy donors (*n* = 18) and patients with pre-diabetic conditions (*n* = 41), T2DM (*n* = 39), or type 3c diabetes (T3cD, pancreatogenic diabetes, *n* = 35). *TAAR1* expression was detected in all study groups (Figure 5a), while other *TAARs* were not expressed or expressed below the cut-off (this discrepancy with data discussed above may be because of the methodological aspects that impact sequencing sensitivity). 

Considering that co-expressed genes are implicated in the same functions [174], we estimate the functional similarity of genes co-expressed with *TAAR1* in healthy controls and patients with prediabetic conditions or diabetes. GO biologic process terms semantic similarity was used to quantify the functional association of *TAAR1* co-expressed (r > 0.5, *p* < 0.05) genes. The semantic similarity was estimated by the Wang coefficient [175] by applying the GOSemSim [176] R package version 2.26.1 in each gene cluster. Then, the Wang coefficient values were compared between study groups by a Wilcoxon signed rank test. A random 400-gene set was included in the comparison.

We found that co-expressed genes in healthy donors have significantly higher (*p* < 0.001) functional relationships compared to prediabetic, T2D, and T3cD groups (Figure 5b). Also, both in healthy donors and pre-diabetic patients, the functional similarity of *TAAR1* co-expressed genes was higher (*p* < 0.001) than in the random gene set. The dramatic loss of functional similarity between *TAAR1* co-expressed genes was identified in pancreatic islets of T2D patients, and, especially, in pancreatogenic T3cD patients. This loss of functional links may be related to the disturbance of TAAR1-mediated biologic processes with diabetic-associated islets’ damage.

### 8.3. RNA Sequencing of Single Cells and Cell Fractions Demonstrates Taar Expression in all Main Islet Cell Populations, Especially in δ-Cells

Analysis of single-cell RNA sequencing (scRNA-seq) data and data obtained by RNAseq in islet cell fractions confirms that *TAAR1* expression is observed in the δ-cells of the pancreatic islets (Table 3). According to the dataset GSE81547, TAAR1 is expressed in 7% of the human pancreatic δ cell population.

*TAAR1* mRNA was identified in other pancreatic cell populations, including α-cells. Analysis of other scRNA-sec datasets and transcriptomic datasets of cell fractions (GSE86469, GSE73727, GSE57973) also revealed *TAAR1* expression in β-cells and PP cells of pancreatic islets, and duct cells belonging to the exocrine pancreas.

Thus, the expression of *TAAR1* in islets is associated with δ-cells, while the expression of other TAARs in the endocrine pancreas cells seems to be sporadic. δ-cells are SST-producing cells. In human islets, the SST-secreting δ-cells make up 1–5% of the total islet cell population [177]. These cells express receptors that can sense appetite-regulating hormones leptin (LEPR) and ghrelin (GHSR), the growth factor neuregulin 4 (ERBB4), and the DA (D2R). The function of δ-cells is the integration of complex metabolic signals via different signaling pathways and regulation of insulin and glucagon synthesis in β- and α-cells, respectively [25]. It is possible that TAAR1 activation in δ cells causes the effect that then becomes amplified downstream.

## 9. Review Limitations

There are several limitations in this review that need to be acknowledged. Current knowledge of the trace amine and trace amine-associated function regulatory functions remains fragmental and needs further studies. The agonists of TAAR2, TAAR6, TAAR8, and TAAR9 are not well defined, and high-specific agonists are not available for these receptors. TAARs are considered rheostats of GPCR-mediated monoamine signaling, but their impact on the other GPCR-mediated pathways has not been studied. Thus, it is difficult to identify the biological base of some identified trends.

As the literature data for TAAR expression in pancreatic islet and islet cell populations are limited, we add the information mined from the GEO NCBI repository. It should be noted that TAARs are expressed at low levels, thus only transcriptomic datasets that were generated with suitable sequencing depth are suitable for the estimation of these receptors’ expression. Therefore, the lack of TAAR expression in some samples may be because of false-negative results related to the insufficient sequencing depth. The RNA sequencing data from different groups cannot be completely standardized and combined for statistical analysis. We can only demonstrate some similar trends that are reproduced in different datasets and find out that the result is congruent and replicable.

The results generated by the systems biology methods like the semantic similarity analysis and GO terms or KEGG pathway enrichment analysis need further confirmation by the laboratory studies.

## 10. Conclusions

Currently, the concept of TAs and their receptors is gradually expanding outside their function in the olfactory system. TAAR1 primarily was shown outside the olfactory system, and this receptor has already been identified as the target of therapeutic agents for schizophrenia, depression, and drug addiction. Its role in the pathogenesis of other diseases, including MetS, continues to be studied. The pattern of TAAR expression allows suspecting the involvement of the TA system in the MetS pathogenesis. Other TAARs were also found in endocrine glands that control energy metabolism, including the hypothalamus, thyroid, and pancreatic islets. In addition, TAARs are important endogenous modulators of other neurotransmitter systems, in particular, dopaminergic transmission, which can be disturbed in MetS. Also, the antagonistic relationship between DA and insulin has been described. A deficiency in DA neurotransmission could cause MetS or its individual symptoms, including weight gain, insulin resistance, hypertension, and glucose intolerance. Meanwhile, insulin deficiency may trigger the development of depression and eating behavior disorders. DA signaling in the endocrine pancreas regulates insulin secretion and maintains glucose homeostasis. TAAR1 ligands in physiological concentration negatively regulate the DA system, which may draw attention to these compounds as putative therapeutic agents for MetS prevention or treatment. However, this hypothesis is contradicted by the effect of some natural TAAR1 agonists, which can elicit insulin resistance. Such results may be explained by the difference in exposure, dose, or the complex interaction of the TA system with other regulatory pathways.

By using bioinformatics approaches, we observed TAAR expression in pancreatic islets. Our in silico analysis demonstrates that TAARs, especially TAAR1, may be involved in the complex regulation of GSIS in the response to chemical stimuli, which is destroyed in type 2 diabetes and prediabetic metabolic disorders. Furthermore, TAARs were identified not only in insulin-producing β-cells but also in α- and δ-cells, which are involved in the GSIS regulation. The effect of TAAR ligands on these cells, especially on the δ-cells, has not yet been investigated. These facts, along with the reported effects of TAAR1 agonists, should be considered in future research to explore if MetS management with TAAR-targeting compounds is possible.

## Figures and Tables

**Table 1:** TAAR genes diversity in human and animal models.

Species	Number of Genes	Identified TAAR Genes
*Homo sapiens*	6	*TAAR1*, *TAAR2*, *TAAR5*, *TAAR6*, *TAAR8*, *TAAR9*
*Pan trogloditus*	3	*TAAR1*, *TAAR5*, *TAAR6*
*Chlorocebus sabaeus*	4	*TAAR1*, *TAAR2*, *TAAR5*, *TAAR6*
*Mus musculus*	15	*Taar1*, *Taar2*, *Taar3*, *Taar4*, *Taar5*, *Taar6*, *Taar7a*, *Taar7b*, *Taar7d*, *Taar7e*, *Taar7f*, *Taar8a*, *Taar8b*, *Taar8c*, *Taar9*
*Rattus norvegicus*	18	*Taar1*, *Taar2*, *Taar3*, *Taar4*, *Taar5*, *Taar6*, *Taar7a*, *Taar7b*, *Taar7c*, *Taar7d*, *Taar7e*, *Taar7f*, *Taar7h*, *Taar7g*, *Taar8a*, *Taar8b*, *Taar8c*, *Taar9*

**Table 2:** Whole tissue sample transcriptome datasets included in analysis.

Accesion	Title	*n*	TAAR1	TAAR2	TAAR5	TAAR6	TAAR8	TAAR9
GSE108072	Global transcriptomic analysis of human pancreatic islets (RNAseq)	88	0.03–1.78 *	0.01–0.06	0.01–0.07	0.01–0.06	0.01–0.05	0.01–0.06
GSE165121	Transcriptional analysis of islets of Langerhans from organ donors of different ages	30	0.8–28,6	0–0.1	0–18.9	0–10.5	0.5–18	0–10.6
GSE50244	Global transcriptomic analysis of human pancreatic islets reveals novel genes influencing glucose metabolism (RNA-seq)	89	0–0.03	NA	NA	NA	NA	NA
GSE164416	Multi-omics profiling of living human pancreatic islet donors reveals heterogeneous beta cell trajectories toward type 2 diabetes	133 **	0–4.8	0–0.2	0–0.9	0–0.2	0–0.3	0–0.9
GSE86468	Single-cell transcriptomics defines human islet cell signatures and reveals cell-type-specific expression changes in type 2 diabetes (bulk)	24	0	0	0	0	0	0

* All expression values are represented in CPM. ** The study includes three groups, i.e., 18 healthy controls, 41 patients with impaired glucose tolerance, 35 type 3c diabetic patients, and 39 type 2 diabetic patients.

**Table 3:** Single-cell RNA seq and cell fractions transcriptome datasets included in the analysis.

Accession	Title	Cells	*n*	TAAR1	TAAR2	TAAR5	TAAR6	TAAR8	TAAR9
GSE57973	Age-dependent gene expression changes in human islets	α-cells	10	0–0.2 *	ND	0–0.07	0–0.08	0–0.08	0–0.04
		β-cells	10	0–1.14	0–0.4	0–0.96	0–0.97	0–0.2	0–0.04
GSE81547	Single-cell transcriptome analysis of human pancreas reveals transcriptional signatures of aging and somatic mutation patterns.	α-cells	998	0–2.25	0	0	0	0–111	0–0.79
		β-cells	348	0	0	0	0	0	0
		δ-cells	83	0–173	0	0	0	0	0
GSE80780	Human islets contain four distinct subtypes of β cells	β-cells	32	0–0.26	0–0.06	0–0.6	0	0–0.06	0–0.08
GSE86469	Single-cell transcriptomics defines human islet cell signatures and reveals cell-type-specific expression changes in type 2 diabetes (single-cell)	α/β/δ/stellate cells	239/263/25/19/18	0	0	0	0	0	0
		PP	18	0–0.8	0	0	0	0	0
GSE73727	Single-cell transcriptomics reveals unique features of human pancreatic islet cell subtypes	α-cells	18	0.1–0.54	0.3–1.6	0.1–0.54	0.1–0.54	0.1–0.54	0.1–0.54
		β-cells	12	0.08–0.46	0.24–1.37	0.08–0.46	0.08–0.46	0.08–0.46	0.08–0.46
		δ-cells	2	0.18–0.24	0.55–0.71	0.19–0.23	0.18–0.024	0.18–0.024	0.18–0.24
		PP cells	9	0.1–0.58	0.3–1.74	0.1–0.58	0.01–0.58	0.01–0.58	0.01–0.58
GSE106148	α Cell Function and Gene Expression Are Compromised in Type 1 Diabetes	α-cells	5	0–1	0–1	0	0	0	0

* All expression values are represented in CPM.

## Data Availability

All datasets are available in the GEO database (https://www.ncbi.nlm.nih.gov/geo/, accessed on 15 September 2023).
